# Editorial Notes and Miscellany

**Published:** 1912-03

**Authors:** 


### Editorial Notes and Miscellany.

An Experienced Prescription Druggist wants a position. Address this office, Texas Medical Journal.

---

The Great Lister is Dead.—Dispatches from London, February 10th (ult.) announce the death of Sir Joseph (Lord) Lister, aged 84 years. Gone to join the immortals.

---

"The Strange Case of Dr. Bruno" ($1.50) will be given as a premium for 2-years' subscription to the "Red Back." Renewals count, if arrears are paid. $1.50+$2.00=$3.50—for $2.00, or for $3.00 3-years' subscription and the premium.

---

Dr. J. R. Papin, representing G. W. Carnrick & Co. (Anti-Thermoline), New York, favored the office with a call recently. We take pleasure in commending him to our readers as a gentleman worthy of their courtesy and regard. He represents first-class products that it will be worth while to investigate.

---

The Hookworm in Texas.—Dr. C. W. Stiles, a Marine Hospital surgeon, has made an extensive canvass in Texas, and reports to the Texas State Health Department that he found hookworm in forty-six counties in the State, and of 1776 school children inspected he found 46 per cent "defective—physically." He thinks medical inspection of schools would remedy much of this.

---

THE EDITOR wishes to direct attention to the card of Mr. Anton Luthy, the Swiss masseur of Austin, and to add his personal endorsement. Mr. Luthy solicits employment by physicians who have occasion to recommend massage in their practice.

---

AFTER THIRTEEN YEARS constant reading the "Red Back," I find that I have "the habit," and can not do without it. Luck to the "Red Back" and its editor.

Sincerely,

Elysian Fields, Texas. L. P. TENNEY.

---

CLEAN UP is the slogan in Texas. *Holland's Magazine* offers three prizes—$200, $300 and $500—for the towns in Texas that can make the best showing in 1912 for cleanliness. Sanitation is on the boom. For particulars, drop a card to the editor, Dallas, Texas. We acknowledge the courtesy of an invitation to meet others in conference as guests of the wide-awake publishers of the wide-awake *Holland's*.

---

THE *New York Medical Times* changes hands, and Dr. H. S. Baketel becomes editor-in-chief. Romaine Pierson, publisher of the *Practical Druggist*, of New York, has purchased from Dr. Alfred Kimball Hills the *Medical Times*, a publication just closing its fortieth year. Mr. Pierson has engaged Dr. H. S. Baketel as editor-in-chief of the *Times*.

A better selection could not have been made.—D.

---

TEXAS TO MANUFACTURE ANTITOXINES.—The State Health Department has begun the establishment at Austin of a laboratory for the manufacture of diphtheria antitoxin, meningitis antitoxin, typhoid antitoxin, etc., the Attorney General having decided that State money can be used for that purpose. The cost of these several sera is so exorbitant that they are out of the reach of the poor, costing, as they do, from $5.00 to $9.00 per "dose." It is said by the Texas Health people that they can be furnished for 50 to 75 cents, and they will be distributed at cost price.

CONFERENCE ON TUBERCULOSIS.—The Governor of Texas has issued a call for a conference on tuberculosis to be held at Waco, April 16th and 17th (prox.). The Governors of Arizona, California, Colorado, Oklahoma, Kansas, Nevada, New Mexico and Utah have been requested to be present, and to appoint delegates to represent their respective States and to request mayors of the larger towns to do the same. The purpose is to discuss ways and means to provide hospital facilities for indigent consumptives,—to corral the floating "carriers" of the germ,—a constant menace to the public. It is thus not so much a charitable or humanitarian move as one of preventive measures in the interest of the public health. Doubtless the discussion will take a wide range and embrace other phases of sanitation. It is hoped the medical profession will take an active part,—although they have not, so far, been recognized by His Excellency; the trustees or managers of the Tuberculosis Sanitaria in Texas all being laymen.

